# Restoration of Cullin3 gene expression enhances the improved effects of sonic hedgehog signaling activation for hypertension and attenuates the dysfunction of vascular smooth muscle cells

**DOI:** 10.1186/s12938-022-01002-w

**Published:** 2022-06-17

**Authors:** Jian Shen, Youqi Li, Menghao Li, Zhiming Li, Huantang Deng, Xiongwei Xie, Jinguang Liu

**Affiliations:** 1 Department of Cardiology, Huizhou Municipal Central Hospital, 41 Eling North Road, Huizhou, 516001 Guangdong China; 2 Department of Nephrology, Huizhou Municipal Central Hospital, Huizhou, 516001 Guangdong China

**Keywords:** CUL3, Vascular smooth muscle cells, Hypertension, Proliferation, Migration, Sonic hedgehog

## Abstract

**Background:**

Hypertension is known as a major factor for global mortality. We aimed to investigate the role of Cullin3 (CUL3) in the regulation of hypertension.

**Material and methods:**

Human vascular smooth muscle cells (VSMCs) were treated with Angiotensin II (Ang II) to establish a hypertension in vitro model. Cell viability was detected by a cell counting kit-8 (CCK-8) assay. The content of reactive oxygen species (ROS) was evaluated by kit. Transwell assay and TUNEL staining were, respectively, used to assess cell migration and apoptosis. Additionally, the expression of sonic hedgehog (SHH) signaling-related proteins (SHH, smoothened homolog (Smo) and glioblastoma (Gli)) and CUL3 was tested with western blotting. Following treatment with Cyclopamine (Cycl), an inhibitor of SHH signaling, in Ang II-induced VSMCs, cell viability, migration, apoptosis and ROS content were determined again. Then, VSMCs were transfected with CUL3 plasmid or/and treated with sonic hedgehog signaling agonist (SAG) to explore the impacts on Ang II-induced VSMCs damage. In vivo, a hypertensive mouse model was established. Systolic blood pressure and diastolic blood pressure were determined. The histopathologic changes of abdominal aortic tissues were examined using H&E staining. The expression of SHH, Smo, Gli and CUL3 was tested with western blotting.

**Results:**

Significantly increased proliferation, migration and apoptosis of VSMCs were observed after Ang II exposure. Moreover, Ang II induced upregulated SHH, Smo and Gli expression, whereas limited increase in CUL3 expression was observed. The content of ROS in Ang II-stimulated VSMCs presented the same results. Following Cycl treatment, the high levels of proliferation and migration in Ang II-treated VSMCs were notably remedied while the apoptosis and ROS concentration were further increased. Moreover, Cycl downregulated SHH, Smo, Gli and CUL3 expression. Above-mentioned changes caused by Ang II were reversed following SAG addition. Indeed, SAG treatment combined with restoration of CUL3 expression inhibited proliferation, migration, apoptosis and ROS level in Ang II-stimulated VSMCs. In vivo, SAG aggravated the histopathological changes of the aorta and with a worse tendency after both SAG intervention and CUL3 silencing. By contrast, SAG treatment and rebound in CUL3 expression alleviated the vascular damage.

**Conclusions:**

Collectively, restoration of CUL3 gene expression protected against hypertension through enhancing the effects of SHH activation in inhibition of apoptosis and oxidative stress for hypertension and alleviating the dysfunction of VSMCs.

## Introduction

Hypertension is publicly recognized as a major factor for global mortality as it has affected more than 25% of the world’s population and is expected to rise to 30% by 2025 [1]. Hypertension is the most important predisposing contributor to cardiovascular diseases that can lead to pathological change in multiple organs [2, 3]. This wide-spread public health issue is striking more and more youngsters currently. Recently, although extensive efforts have been performed to improve the treatment rate for hypertension, the treatment status is unsatisfactory [4, 5]. Therefore, more studies are of paramount importance and necessity to identify the pathogenesis and develop new strategies and targets to effectively relieve hypertension.

A growing body of evidence suggests that aberrant vascular smooth muscle cells (VSMCs) proliferation, migration and apoptosis are major pathological phenomena in the development of hypertension [6, 7]. Angiotensin (Ang) II, a vasoactive peptide in the renin–angiotensin system, constricts blood vessels and increases blood pressure through its structural and functional effects, thereby provoking the pathological development of hypertension via increasing blood pressure [8]. Ang II stimulation can promote the proliferation and influence migration and apoptosis of VSMCs [9]. Cullin3 (CUL3) is a crucial subunit of the CUL3-Ring-Ligase (CRL3) ubiquitin ligase complex that serves as a molecular scaffold linking the E3 ubiquitin ligase Rbx1 to adaptors carrying protein substrates [10, 11]. It has been well reported that mutations in the CUL3 are the direct cause of pseudohypoaldosteronism type II (PHAII), which results in hypertension and hyperkalemia [12]. CUL3 exerts a pivotal role in maintaining the balance of arterial pressure, and loss of its ubiquitin ligase activity is closely implicated in hypertension [13, 14]. Autosomal dominant mutations in CUL3 cause the most severe form of familial hyperkalemic hypertension [15]. A recent study shows the levels of CUL3 and its neddylated derivatives were substantially increased in the aortic tissues and heart of the streptozotocin-induced mice, and dysfunction of CUL3 RING E3 ubiquitin ligase caused vasoconstriction and increased sodium reabsorption in diabetes mice [16]. Report has demonstrated previously that CUL3 negatively regulates the stability of sonic hedgehog (SHH) signaling through ubiquitination degradation of glioblastoma (Gli), an important intracellular factor involved in SHH signal transduction [17]. Activation of the SHH signaling can improve Ang II-induced hypertension by enhancing nitric oxide (NO) release and reducing oxidative stress in the vessel wall [18]. However, the relation and balance between CUL3 and SHH signaling in hypertension draw our research interests.

In the current study, our study aimed to prove that restoration of CUL3 gene expression relieved hypertension through enhancing the effects of SHH activation in inhibition of apoptosis and oxidative stress as well as attenuating the proliferation and migration of VSMCs. These findings may provide a novel therapeutic target for hypertension and be of critical significance for the experts to disclose the potential pathogenesis of hypertension.

## Results

### Ang II stimulation promotes proliferation, migration, oxidative stress and apoptosis of VSMCs as well as activates SHH signaling

**Fig. 1:**
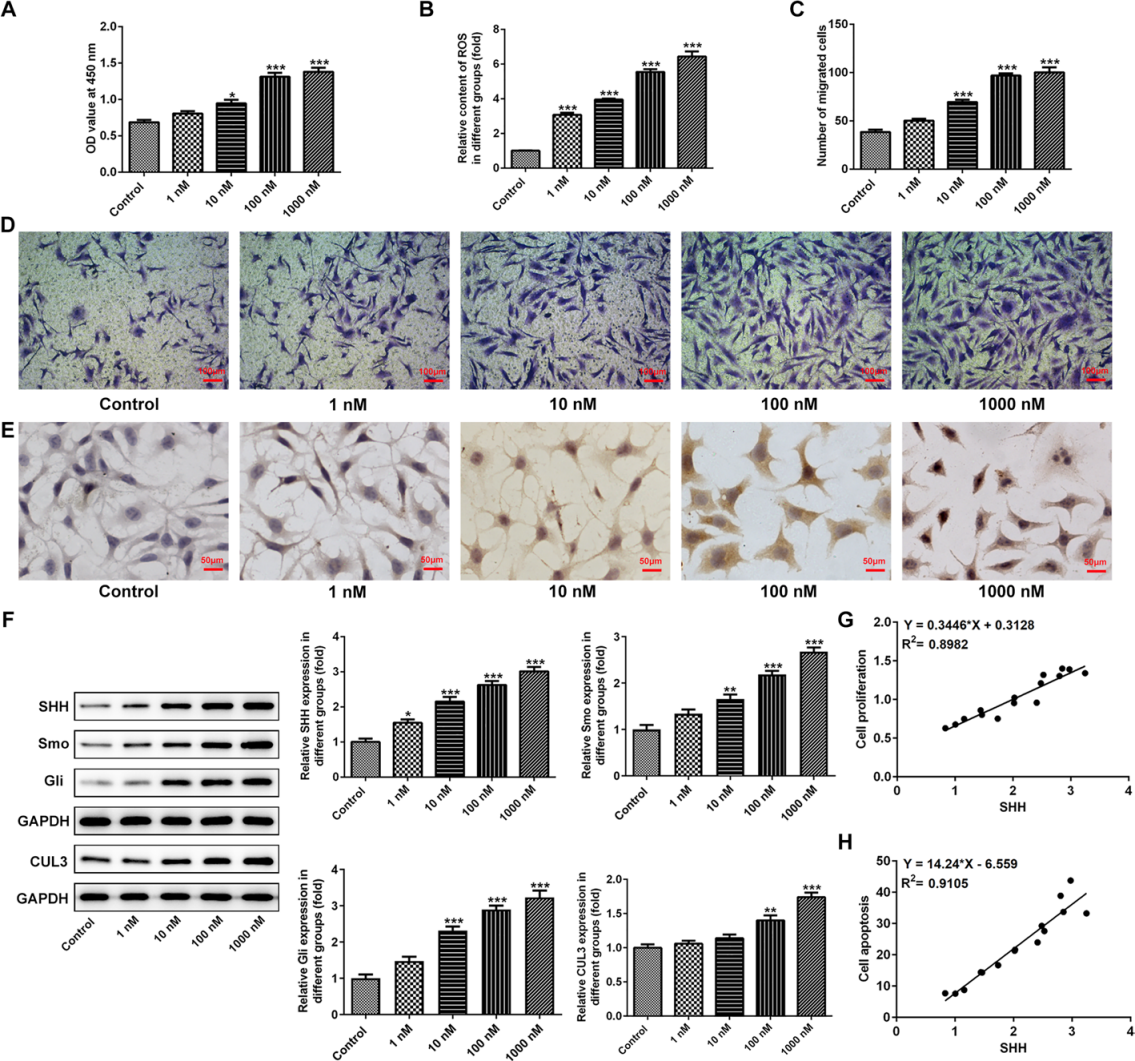
Ang II stimulation promoted proliferation, migration, oxidative stress and apoptosis of VSMCs as well as activated SHH signaling. **A** Cell viability was measured by a CCK-8 assay. **B** ROS content was evaluated using the commercial kit. **C**,** D** Detection of cell migration using Transwell migration assay. Magnification, × 100. **E** TUNEL staining was adopted for the determination of cell apoptosis. Magnification, × 200. **F** Analysis of protein expression related to SHH signaling by western blot analysis. **G** The correction between SHH expression and cell proliferation. **H** The correction between SHH expression and cell apoptosis. *P < 0.05, **P < 0.01, ***P < 0.001 vs. control

Ang II stimulation can promote the proliferation and influence migration and apoptosis of VSMCs [9]. VSMCs were treated with Ang II to simulate the hypertension model in vitro. Firstly, cell viability was evaluated by a CCK-8 assay after Ang II stimulation. As shown in Fig. 1A, Ang II led to a dose-dependent increase in the viability of VSMCs compared to the control group. Results presented in Fig. 1B indicated that the level of ROS was significantly enhanced in the Ang II-treated group when compared to the untreated group. Additionally, notably elevated ability of cell migration was observed in VSMCs under Ang II condition (Fig. 1C, D). Besides, the number of apoptosis cells was markedly increased in VSMCs exposed to Ang II (Fig. 1E). Western blot analysis was used to determine the expression of proteins in SHH signaling pathway. As observable from Fig. 1F, the expression of SHH, Smo, Gli and CUL3 was apparently upregulated in the Ang II groups in a dose-dependent manner relative to the control group. Subsequently, the correction between cell proliferation or cell apoptosis and SHH expression was analyzed using Pearson correlation coefficient. As displayed in Fig. 1G, H, SHH expression level is positively correlated with cell proliferation and apoptosis. 100 nM Ang II was selected for conduct the subsequently experiments. These results suggest that SHH signaling is closely related to the VSMC cell injury induced by Ang II.

### Inhibition of SHH signaling regulates the proliferation, migration, oxidative stress and apoptosis of VSMCs exposed to Ang II

**Fig. 2:**
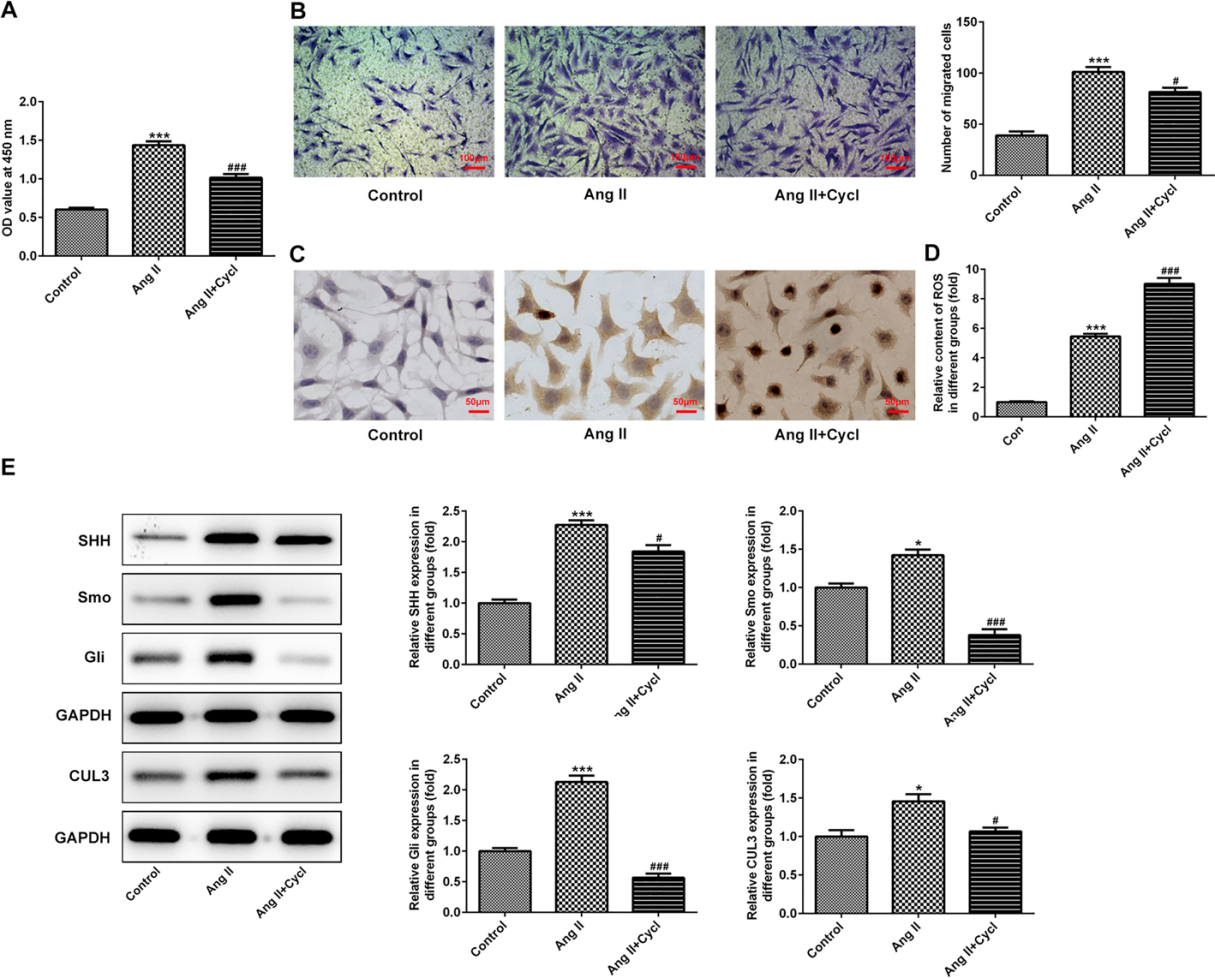
Inhibition of SHH signaling affected the proliferation, migration, oxidative stress and apoptosis of VSMCs induced by Ang II. **A** Assessment of cell viability with a CCK-8 assay. **B** Determination of cell migration using Transwell migration assay. Magnification, × 100. **C** Analysis of cell apoptosis by TUNEL staining. Magnification, × 200. **D** The level of ROS was examined with a commercial available kit. **E** Expression of proteins related to SHH signaling was evaluated with western blot assay. *P < 0.05, *P* < 0.001 vs. control; ^#^*P* < 0.05, ^###^*P* < 0.001 vs. Ang II

Then, Cycl, an inhibitor of SHH signaling, was used to treat VSMCs under 100 nM Ang II condition. It was found that Cycl reduced proliferation and migration but enhanced apoptosis and ROS level of VSMCs when compared to the Ang II group (Fig. 2A–D). Results of western blot presented in Fig. 2E indicated that the expression of SHH, Smo, Gli and CUL3 was significantly downregulated in Ang II + Cycl group. Above data demonstrate that inhibition of SHH signaling can affect the proliferation, migration, oxidative stress and apoptosis of VSMCs exposed to Ang II.

### Upregulation of CUL3 expression combined with activation of SHH signaling protects against Ang II-induced VSMCs damage

**Fig. 3:**
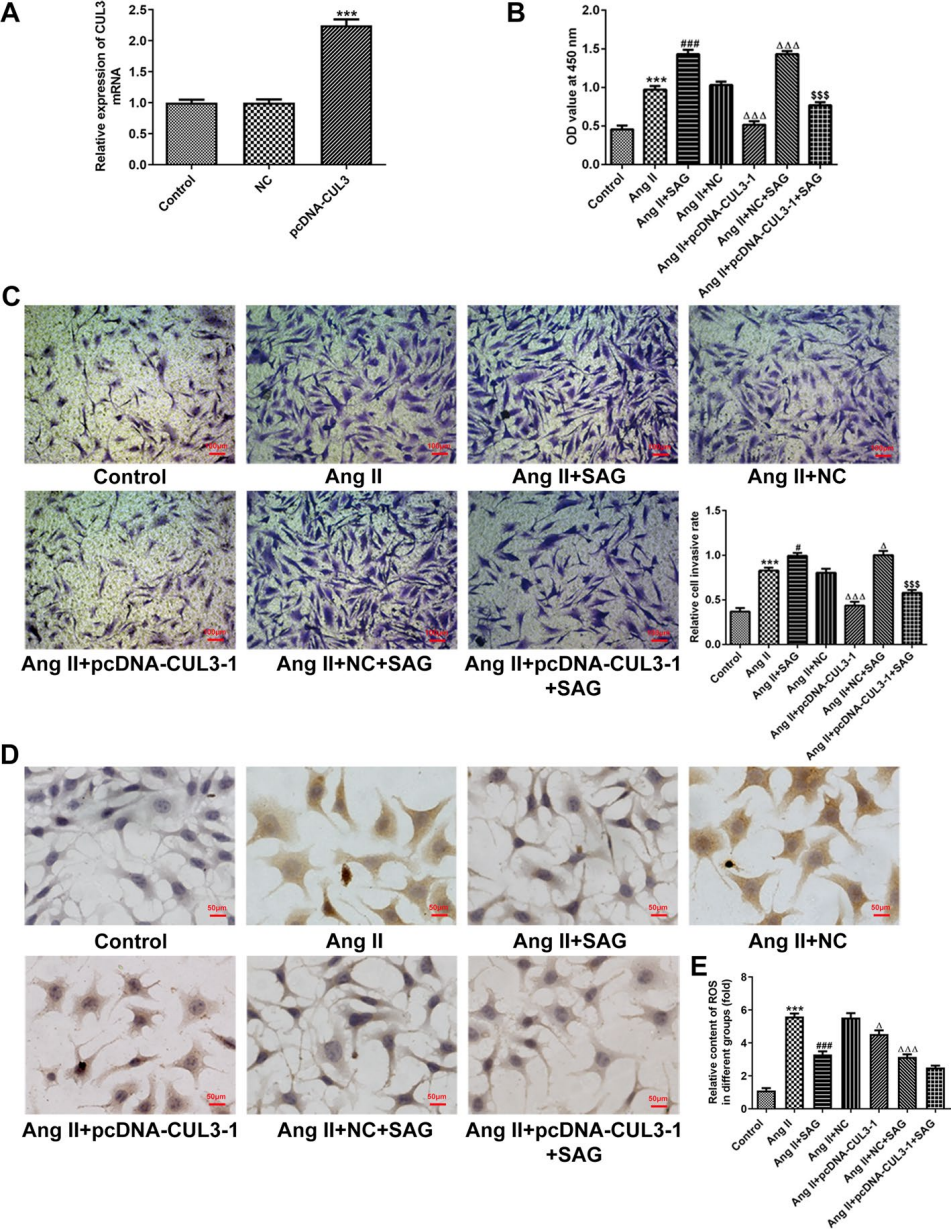
Upregulation of CUL3 expression combined with activation of SHH signaling protected against Ang II-induced VSMCs damage. **A** Detection of CUL3 mRNA expression with RT-qPCR. ^***^P < 0.001 vs. NC. **B** Cell viability was tested with a CCK-8 assay. **C** Measurement of cell migration using Transwell migration assay. Magnification, × 100. **D** Cell apoptosis was assessed by TUNEL staining. Magnification, × 200. **E** ROS level was determined with commercial available kit. ^***^P < 0.001 vs. control; ^#^*P* < 0.05, ^###^*P* < 0.001 vs. Ang II; ^△^*P* < 0.05, ^△△△^*P* < 0.001 vs. Ang II + NC; ^$$$^*P* < 0.001 vs. Ang II + NC + SAG

**Fig. 4:**
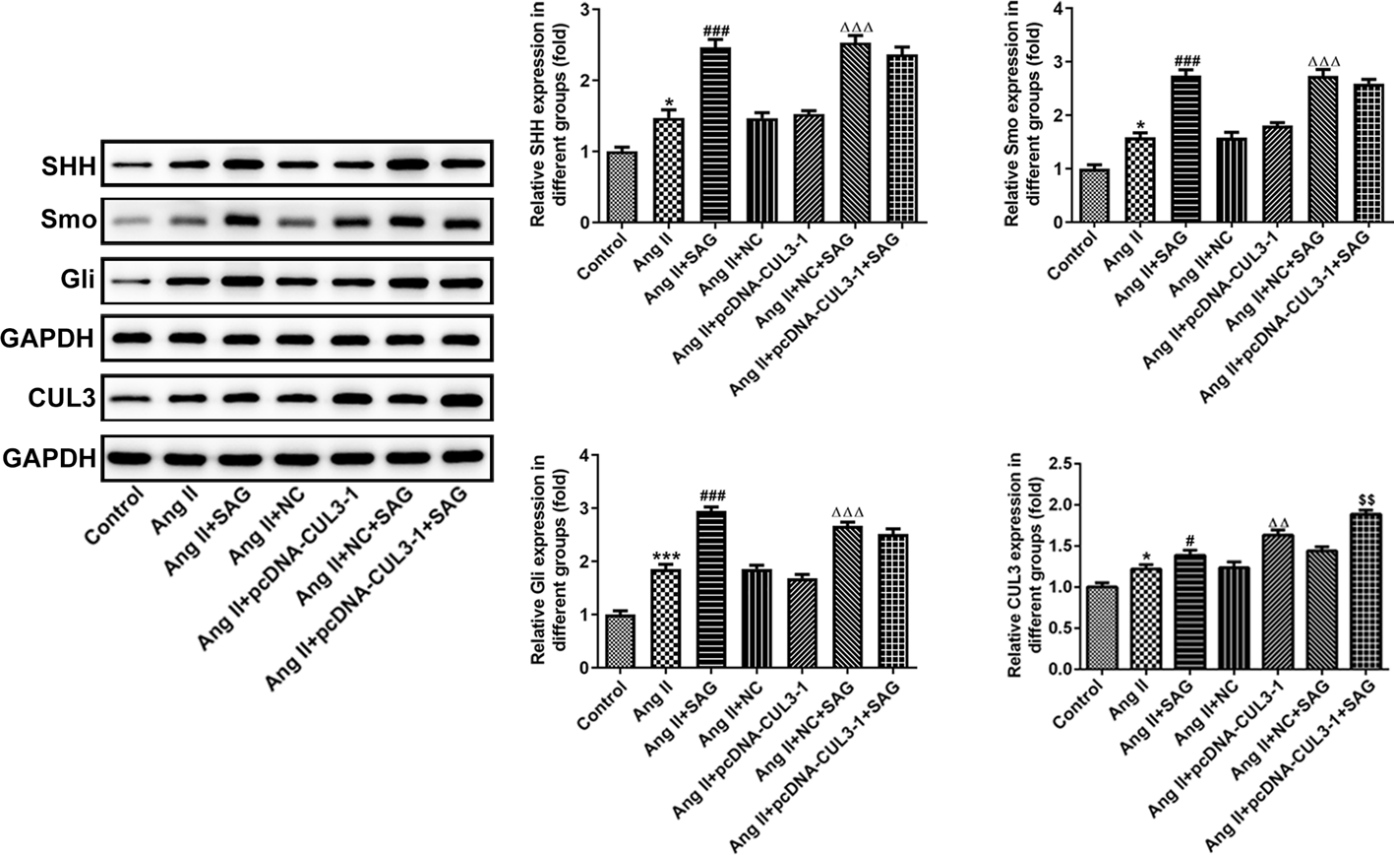
CUL3 overexpression combined with SHH signaling activation regulated the expression of proteins related to SHH signaling in Ang II-induced. Western blot analysis was adopted for the evaluation of SHH, Smo, Gli and CUL3 expression. *P < 0.05, ***P < 0.001 vs. control; ^#^*P* < 0.05, ^###^*P* < 0.001 vs. Ang II; ^△△^*P* < 0.01, ^△△△^*P* < 0.001 vs. Ang II + NC; ^$$^*P* < 0.01 vs. Ang II + NC + SAG

To explore the relation and balance between CUL3 and SHH signaling in Ang II-induced VSMCs, CUL3 was overexpressed by CUL3 plasmid. Significantly upregulated CUL3 mRNA expression was observed in the pcDNA-CUL3 group compared with the empty vector group (Fig. 3A). Then, as shown in Fig. 3B, C, SAG, an agonist of SHH signaling, elevated cell viability and cell migration capacity relative to the Ang II group. In addition, CUL3 overexpression decreased Ang II-induced proliferation and migration of VSMCs. Meanwhile, CUL3 upregulation reversed the impacts of SAG treatment on the proliferation and migration of VSMCs exposed to Ang II. Moreover, the levels of cell apoptosis and ROS content were attenuated after SAG intervention, and CUL3 overexpression alone also decreased apoptosis and ROS as comparison to the Ang II + NC group (Fig. 3D, E). Further results revealed that VSMCs transfected with CUL3 plasmid and treatment with SAG exhibited reduced cell apoptosis and ROS level, but had no significant difference when compared to the Ang II + NC + SAG group. Furthermore, results in Fig. 4 indicated that SAG notably elevated SHH, Smo, Gli and CUL3 expression relative to the Ang II group. Additionally, pcDNA-CUL3 transfection conspicuously increased CUL3 protein expression when compared to the Ang II + NC group, and further elevated CUL3 expression was found in the Ang II + pcDNA-CUL3 + SAG group. Overall, these data suggest that SAG treatment combined with restoration of CUL3 gene expression inhibits proliferation, migration, apoptosis and ROS levels in Ang II-stimulated VSMCs.

### Upregulation of CUL3 expression combined with activation of SHH signaling alleviated vascular injury of hypertension mouse model induced by Ang II

**Fig. 5:**
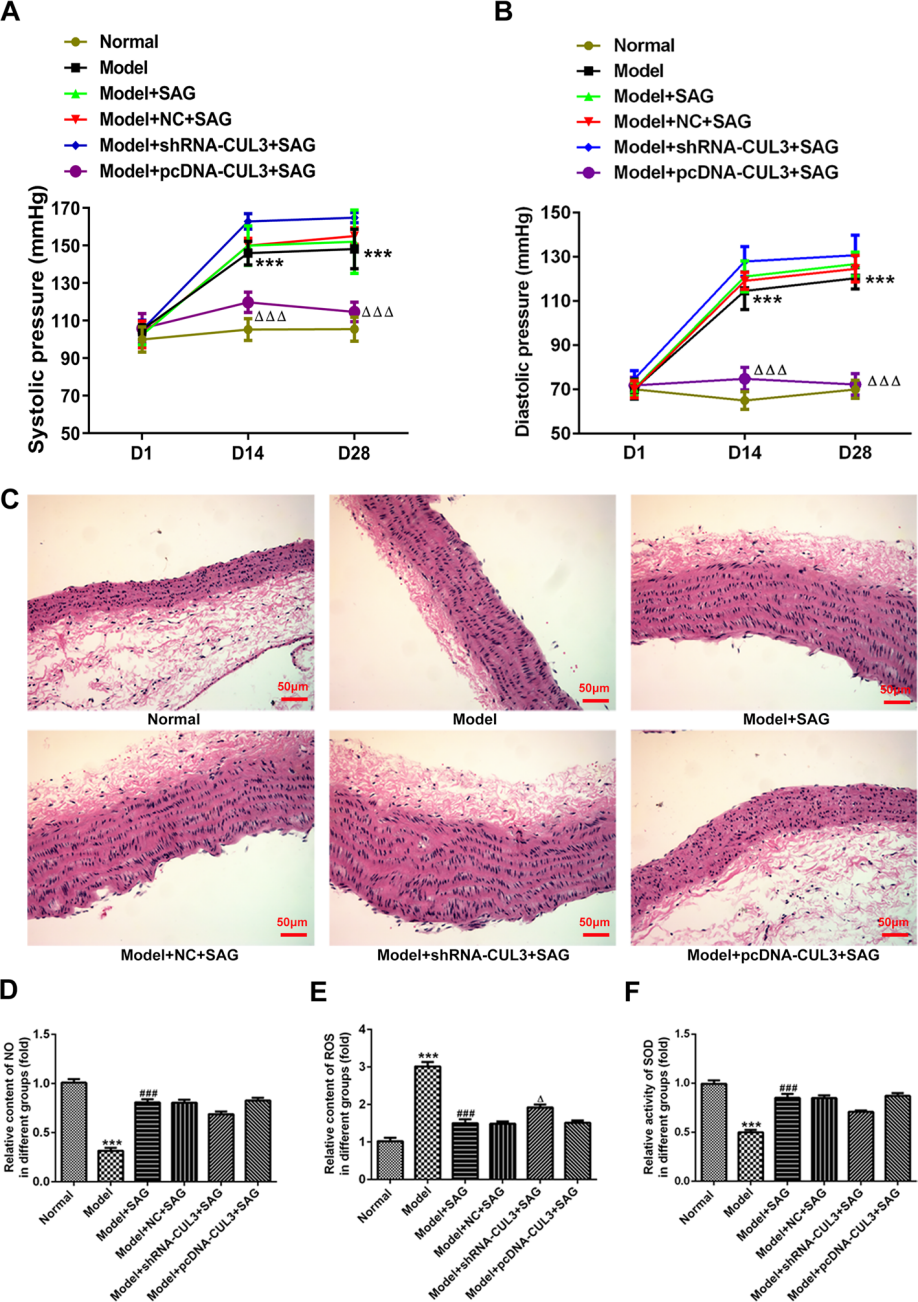
Upregulation of CUL3 expression combined with activation of SHH signaling alleviated vascular injury of hypertension mouse model induced by Ang II. **A**,** B** Measurement of Systolic blood pressure (SBP) and diastolic blood pressure (DBP) at D14 and D28 following insertion of Ang II osmotic pumps by using a tail-cuff blood pressure instrument. **C** The pathological changes of abdominal aortic tissues were evaluated with H&E staining. Magnification, × 200. The levels of **D** NO, **E** ROS and **F** SOD were examined with the corresponding commercial available kit. ***P < 0.001 vs. normal; ^###^*P* < 0.001 vs. model; ^△^*P* < 0.05, ^△△△^*P* < 0.001 vs. model + NC + SAG

**Fig. 6:**
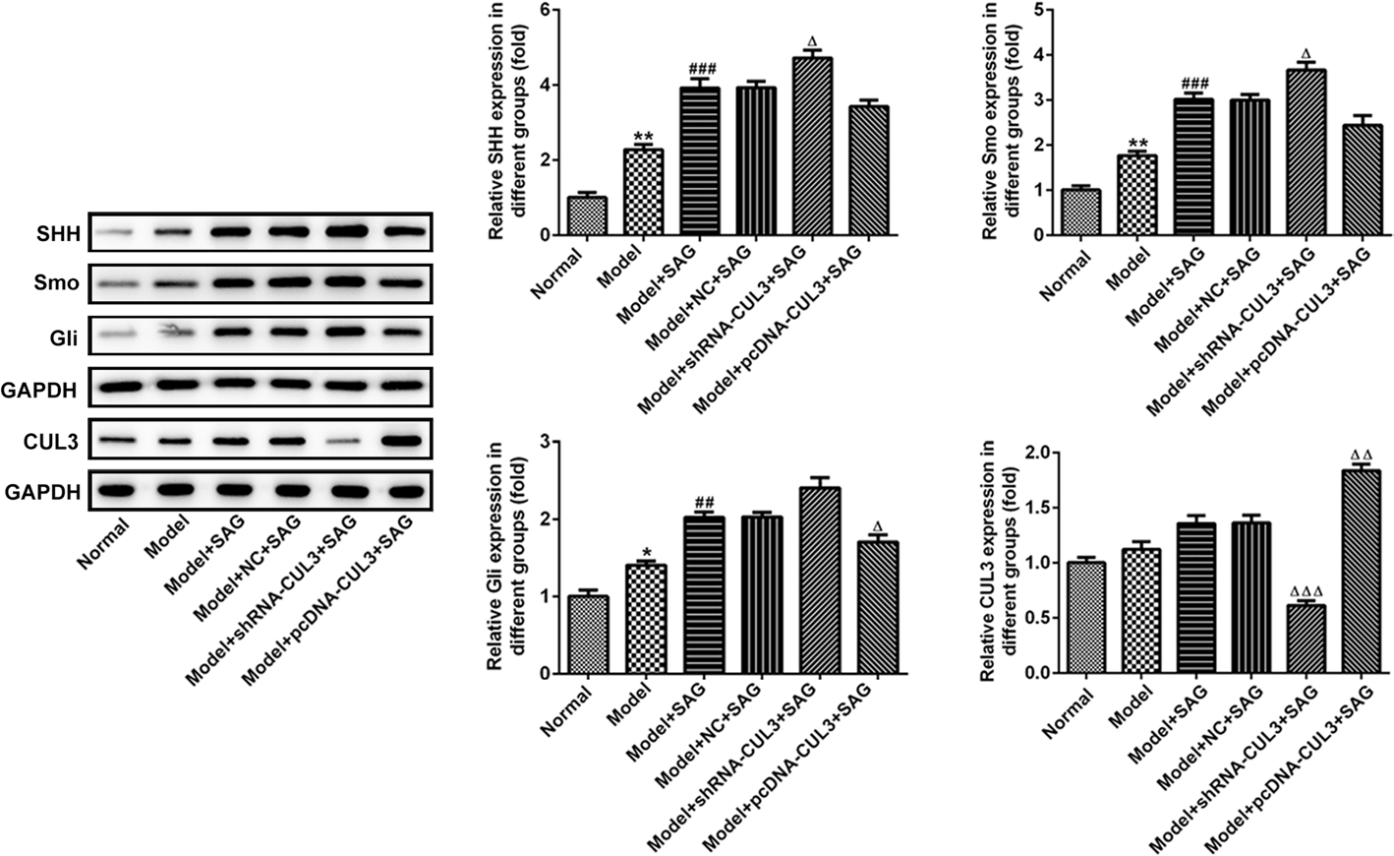
Upregulation of CUL3 expression combined with activation of SHH signaling affected the expression of proteins related to SHH signaling in hypertension mouse model induced by Ang II. Western blot analysis was utilized for the detection of SHH, Smo, Gli and CUL3 expression in abdominal aortic tissues in each group. *P < 0.05, **P < 0.01 vs. normal; ^##^*P* < 0.01, ^###^*P* < 0.001 vs. model; ^△^*P* < 0.05, ^△△^*P* < 0.01, ^△△△^*P* < 0.001 vs. model + NC + SAG

To further investigate the regulatory effects of CUL3 and SHH signaling on hypertension, a hypertension mouse model induced by Ang II was established. The systolic pressure and diastolic pressure were determined 2 and 4 weeks following insertion of Ang II osmotic pumps. As shown in Fig. 5A, B, the levels of systolic pressure and diastolic pressure were notably elevated at Day14 (D14) and D28 in the model group compared to the normal group. Additionally, SAG treatment slightly increased both systolic pressure and diastolic pressure when compared to the model group. The further increasing trend in these two parameters at the two points were found in the model + shRNA-CUL3 + SAG group relative to the model + NC + SAG group. By contrast, CUL3 overexpression combined with SAG treatment significantly reduced systolic pressure and diastolic pressure levels when compared to the model + NC + SAG group. Besides, results of H&E staining indicated that the vascular intima was smoother, with endothelial cells intact in the normal group (Fig. 5C). However, in the model group, the inner membrane was obviously proliferated, the endothelial cells swollen and ruptured, the smooth muscle visibly proliferated, and the outer membrane had a large amount of deposits. Furthermore, the aforementioned cell damage induced by Ang II was more serious in the model + SAG, mode + NC + SAG and model + shRNA-CUL3 + SAG groups. On the contrary, after CUL3 overexpression and SAG treatment, the thickness of the arterial wall was basically uniform, the intima smoother, and the adventitia deposits were significantly reduced relative to the model + NC + SAG group. Moreover, the levels of NO and SOD were obviously decreased while ROS was increased in the model group (Fig. 5D–F). SAG treatment elevated NO and SOD levels but reduced ROS content in abdominal aortic tissues when compared to the model group. ShRNA-CUL3 combined with SAG exhibited decreased tendencies in NO and SOD levels and notably increase in ROS levels as comparison to the model + NC + SAG. However, pcDNA-CUL3 + SAG presented the opposite results with shRNA-CUL3 + SAG. Finally, the expression of SHH, Smo, Gli and CUL3 in abdominal aortic tissues was upregulated in the model group (Fig. 6). SAG further elevated these proteins expression relative to the model group. The further upregulation in the expression of SHH, Smo and Gli were observed in the model + shRNA-CUL3 + SAG group compared to the model + NC + SAG group. By contrast, CUL3 overexpression displayed the opposite effects on the expression of aforementioned proteins with CUL3 silencing. Through the above findings, we proved that upregulation of CUL3 expression together with activation of SHH signaling relieves vascular injury of hypertension mouse model induced by Ang II.

## Discussion

The present study aimed to explore the effects of restoration of CUL3 gene expression on the apoptosis, oxidative stress, proliferation and migration during hypertension as well as its regulation on SHH signaling. Hypertension, whose pathogenesis remains unclear, is a common cardiovascular disease with a high incidence that can trigger more serious diseases such as coronary heart disease, heart failure and renal failure [19, 20]. We demonstrated that restoration of CUL3 expression could relieve hypertension through enhancing the effects of SHH activation on inhibition of apoptosis and oxidative stress as well as attenuating the proliferation and migration of VSMCs. These findings may provide a novel therapeutic target for hypertension.

Fully mature and differentiated VSMCs have obviously plasticity, which is crucial for the maintenance and adaptation of blood vessels. However, the phenotypic regulation of VSMCs is of great significance in the pathobiology of cardiovascular diseases such as hypertension [21]. A growing body of evidence suggests that the proliferation, apoptosis, migration and phenotypic changes of VSMCs are major pathological phenomena in the development of hypertension [22, 23]. Specifically, under the hypertension condition, VSMCs will present high proliferated and apoptotic status [24, 25]. The imbalance between proliferation and apoptosis is an important feature of vascular remodeling [26]. The function of SHH activation, which is to promote proliferation and reduce apoptosis, can improve the pathological conditions in the angiotensin-induced hypertension model [27]. It is noteworthy that SHH can induce NO production and decrease oxidative stress, thereby correcting Ang II-induced hypertension and endothelial dysfunction in aorta [18]. In the present study, our study showed that SHH inhibitor Cycl suppressed proliferation and migration but promoted apoptosis and ROS levels in VSMCs under 100 nM Ang II condition, suggesting a dual effect of SHH signaling on the progression of hypertension.

In the whole exome sequencing study of patients with familial hyperkalemic hypertension, CUL3 was first considered as a candidate gene for this disease [12]. Abnormal protein function caused by CUL3 is associated with atherosclerosis, angina pectoris and acute coronary syndrome, and hypertension itself is a key cause of these diseases [28]. Autosomal dominant mutations in CUL3 causes the most severe form of familial hyperkalemic hypertension [15]. Research has proposed that CUL3 exerts a pivotal role in maintaining the balance of arterial pressure, and loss of its ubiquitin ligase activity is closely implicated in hypertension [13, 14]. Emerging evidence supports the notion that CUL3 might regulate the proliferation, migration and phenotypic transformation of VSMCs in atherosclerosis by modulating Hedgehog signaling pathway [29]. Mutation of CUL3 can lead to vascular smooth muscle defects, leading to severe vascular dysfunction and hypertension [30]. Compelling evidence indicates that CUL3 negatively regulates the stability of SHH signaling through ubiquitination degradation of Gli, an important intracellular factor involved in SHH signal transduction [17]. An increasing number of studies have validated that the regulation of CUL3/Gli balance plays a key role in various physiological and pathological mechanisms hypertension [31, 32]. Therefore, more in-depth research on CUL3 can deepen the understanding of the SHH pathway in hypertension. Our study showed that the expression of CUL3 was increased in VSMCs exposed to Ang II, but compared with the increase of SHH and Gli, the increase of CUL3 was significantly lower than the latter two. de Boer et al., 2018 has demonstrated similar results that CUL3 is associated with atherosclerosis, angina pectoris and acute coronary syndrome, and hypertension itself is a key cause of these diseases [28]. If Gli was overexpressed, insufficient degradation of Gli by CUL3 would lead to excessive proliferation of VSMCs, which would aggravate the pathological conditions of hypertension [32]. From the perspective of reducing the level of apoptosis, elevated expression of CUL3 could reduce the expression of Gli to a normal level, but not restrict the improvement of oxidative stress by SHH and the promotion of NO synthesis in endothelial cells. Therefore, increasing the expression of CUL3 while using SHH activators could not only play an anti-apoptotic and anti-oxidative role in the SHH pathway, but also reduce the level of excessive proliferation and migration of VSMCs, thereby hindering the development of hypertension. Of course, our experiment has a limitation. Due to the length of this paper, we only discussed the effects and regulatory mechanism of CUL3 and SHH signaling in hypertension in vitro and in vivo. We will further investigate the upstream mechanism of CUL3 in hypertension, which is the next experimental plan of our study.

Our findings suggested the importance of the balance between CUL3 and SHH signaling in the progression of hypertension, which provides a new understanding of the regulatory mechanism of this disease. Drugs that can target CUL3 and SHH might have the potential to hypertension therapy in the future.

## Conclusion

To sum up, this study was the first to explore the balance between SHH signaling and CUL3 in the progression of hypertension in vitro and in vivo. We demonstrated that restoration of CUL3 expression could protect against hypertension through enhancing the effects of SHH activation on inhibition of apoptosis and oxidative stress for hypertension and alleviating the dysfunction of VSMCs. This study provides a new understanding for the regulation mechanism of hypertension.

## Materials and methods

### Cell culture and treatment

The human aortic VSMCs were purchased from the American Type Culture Collection (ATCC, Rockville, MD, USA). Cells were cultured in Dulbecco’s modified Eagle’s medium (DMEM, Gibco, USA) supplemented with 10% fetal bovine serum (FBS, Gibco, USA) at 37 °C in a humidified atmosphere of 95% air and 5% CO_2_. VSMCs were seeded in 6-well plates (1 × 10^5^ cells per well). Subsequently, cells were exposed to a series concentrations of Ang II (1, 10, 100 and 1000 nM; Sigma, St. Louis, MO, USA) for 48 h [33]. Cells without Ang II treatment served as the control group. VSMCs were pretreated with Cyclopamine (Cycl; 20 μM), an antagonist of SHH signaling, or sonic hedgehog signaling agonist (SAG; 0.25 μM), an agonist of SHH signaling, for 1 h prior to Ang II treatment [34].

### Cell transfection

Prior to transfection, VSMCs were plated in 6-well plates with the density of 2 × 10^6^ cells in each well. When 60% confluence was reached, transfections of VSMCs with 50 nM overexpressed plasmid which carries CUL3 (pcDNA-CUL3) and 50 nM negative control (NC) for 24 h prior to 100 nM Ang II treatment were conducted using Lipofectamine 2000 reagent (Invitrogen, Carlsbad, CA). Above-mentioned vectors were synthesized by Ribo Bio (Guangzhou, China). Following an incubation period of 24 h, the expression of CUL3 was examined using RT-qPCR.

### Cell counting kit-8 (CCK-8) assay

CCK-8 was applied to determination of cell proliferation. VSMCs were plated in a 96-well plate (5 × 10^3^ cells in each well). Subsequently, 10 μl CCK-8 solution was added to each well of the plate. Following incubation at 37 °C for 1 h, the optical density (OD) was detected using a microplate reader (Lincoln, Nebraska, United States) at 450 nm.

### Transwell migration assay

For the Transwell migration assays, VSMCs were seeded at a density of 1 × 10^5^ cells/well into the upper chambers, containing a filter membrane (8-μm pore size) of 24-well Transwell plates (Corning Inc., New York, USA). DMEM containing 10% FBS was added to the lower chamber. After incubation for 24 h, the non-migrated cells in the upper inserts were removed by a cotton-tipped swab. The migrated cells were fixed with 4% triformol and then stained with 0.1% crystal violet solution. A light microscope was applied to the count of migrated cell number. The images of cell migration were taken with an inverted microscope (BX51; Olympus Corporation, Japan), and the number of migrated cells was calculated.

### Terminal-deoxynucleotidyl transferase-mediated nick end labeling (TUNEL)

The apoptosis of VSMCs was determined using TUNEL assay. In brief, VSMCs were washed with phosphate-buffered saline (PBS) and then fixed with 4% paraformaldehyde. The apoptotic cells were visualized with the TUNEL staining following manufacturer’s recommendations (Beyotime, China). The nuclei of healthy cells were stained blue, while those in apoptotic cells presenting brown/yellow staining which were identified as TUNEL-positive cells. Specimens were mounted and detected using routine light microscopy.

### Quantitative reverse transcription-polymerase chain reaction (RT-qPCR)

Collection of total RNA from VSMCs in this study was used the Trizol reagent (Invitrogen, Waltham, MA, USA). Total RNA was reverse transcribed into complementary DNA (cDNA) using a QuantiTect Reverse Transcription kit (Thermo Fisher Scientific, Inc.). The temperature protocol for this step was as follows: 70 °C for 5 min, 37 °C for 5 min and 42 °C for 1 h. And PCR was carried out using an ABI 7500 FAST system (Applied Biosystems, Foster, CA). The following thermocycling conditions were used: initial denaturation at 95 °C for 10 min; followed by 40 cycles of denaturation at 95 °C for 15 s and annealing at 60 °C for 1 min; and a final extension of 10 min at 72 °C. Specific primers were designed by RiboBio (Guangzhou, China). Primers sequences used in this study were as follows: CUL3, forward 5′-CGAATCTGAGCAAAGGCACG-3′ and reverse 5′-ATCTTCTCGCACCGGAAAGG-3′; glyceraldehyde-phosphate dehydrogenase (GAPDH), forward 5′-AATGGGCAGCCGTTAGGAAA-3′ and reverse 5′-GCGCCCAATACGACCAAATC-3′. The expression of target genes expression was normalized versus GAPDH. The relative fold change of target gene expression was calculated using the 2^−ΔΔCt^ method [35].

### Animal

A total of 60 male Kunming mice (18–22 g) were obtained from Shanghai SLAC Laboratory Animal Company Ltd (Shanghai, China). Mice were maintained in a suitable environment with a 12-h light/dark cycle at 21 ± 3 °C. All animals were allowed free access to tap water and standard chow. Mice were housed for at least one week before the experimental performance. All of the experiments protocols were approved by the Ethics Committee on Animal Experiments of Huizhou Municipal Central Hospital.

### Establishment of mouse hypertension model

The hypertensive mouse model was generated by Ang II infusion using osmotic pumps as described in the previous studies [36, 37]. After anesthesia with sodium pentobarbital intraperitoneally, micro-osmotic pumps were subcutaneously implanted into the back of the mice, allowing continuous delivery of Ang II (1 mg/kg/day) for a consecutive 4 weeks. Mice were divided into six groups and ten mice were included in each group. Mice in the normal group underwent the same surgery without the pump insertion. A regular chow diet was used to feed all experimental subjects. For SAG-treated groups, mice were administered SAG (5 mg/kg) intraperitoneally every day for two weeks. For transfection-treated groups, mice were injected via caudal vein with NC, plasmid or lentivirus, respectively. Entranster™-in vivo transfection reagent (Engreen Biosystem Co, Ltd. China) was employed to deliver the plasmids.

### Noninvasive blood pressure measurement

Systolic blood pressure and diastolic blood pressure (mmHg) were determined 2 or 4 weeks following insertion of Ang II osmotic pumps by a tail-cuff blood pressure instrument (Kent Scientific, Torrington, CT, USA). Prior to start of blood pressure monitoring, mice were introduced daily for 1 week to get used to the system.

### Specimen preparation and staining

Abdominal aortic tissues were harvested, conventionally fixed in 4% paraformaldehyde overnight at 4 °C. Paraffin-embedded posterior segments were then sectioned into 5-μm slices for histological analysis. The sections were deparaffinized with graded ethanol together with xylene. After hematoxylin and eosin (H&E) staining, sections were dehydrated with graded ethanol and xylene according to standard protocol. Images were subsequently obtained using an OLYMPUS DP73 microscope (Olympus Corporation).

### Measurement of reactive oxygen species (ROS), superoxide dismutase (SOD) and NO levels

The levels of ROS, SOD and NO in cell culture supernatants or abdominal aortic tissues were tested by the ROS assay kit, SOD assay kit and NO assay kit according to standard protocols. Aforementioned kits were all provided by Nanjing Jiancheng Bioengineering Institute (Nanjing, China).

### Western blot analysis

Total protein extracted from VSMCs and whole aortas of hypertension mice was lysed in radioimmunoprecipitation (RIPA) lysis buffer (Beyotime, China). And the content of protein was quantified by a bicinchoninic acid (BCA) protein assay kit (Beyotime, China). Then, equal amounts of protein samples were subjected to sodium dodecyl sulfate polyacrylamide gel electrophoresis (SDS/PAGE) and transferred on to polyvinylidene difluoride (PVDF) membranes. 5% skimmed milk was applied to the blockade of these membranes. Subsequently, all membranes were immersed in corresponding primary antibodies (Cell Signaling Technology, Boston, MA, USA) overnight at 4 °C. After rinsing with Tris-buffered saline and Tween 20 (TBST) three times, these membranes were incubated with secondary antibody (Beyotime, China). Finally, the bands were visualized with an enhanced chemiluminescence detection reagent (Pierce, Rockford, USA) and quantified with Image-J software (NIH, USA). GAPDH expression was used as an internal control.

### Statistical analysis

All experiments were repeated three times independently (N = 3) and all data were expressed as the mean ± standard deviation (SD). Data were calculated using GraphPad Prism software, version 8.0 (GraphPad Software Inc., USA). Statistical comparisons between two groups were analyzed using an unpaired Student's t-test, and the comparisons among multiple groups were performed using analysis of variance (ANOVA) followed by Turkey's post hoc test. Statistics were considered significant at *P* value less than 0.05.

## Data Availability

The raw data supporting the conclusions of this article are available from the corresponding author on reasonable request.

## Abbreviations

CUL3: Cullin3
VSMCs: Vascular smooth muscle cells
Ang II: Angiotensin II
SHH: Sonic hedgehog
Gli: Glioblastoma
ROS: Reactive oxygen species
Cycl: Cyclopamine
SAG: Sonic hedgehog signaling agonist
PHAII: Pseudohypoaldosteronism type II
NO: Nitric oxide
DMEM: Dulbecco’s modified Eagle’s medium
FBS: Fetal bovine serum
NC: Negative control
OD: Optical density
PBS: Phosphate-buffered saline
cDNA: Complementary DNA
GAPDH: Glyceraldehyde-phosphate dehydrogenase
H&E: Hematoxylin and eosin
BCA: Bicinchoninic acid
SDS/PAGE: Sodium dodecyl sulfate polyacrylamide gel electrophoresis
PVDF: Polyvinylidene difluoride
SD: Standard deviation
ANOVA: Analysis of variance
